# Chronic Arsenic Exposure and Blood Glutathione and Glutathione Disulfide Concentrations in Bangladeshi Adults

**DOI:** 10.1289/ehp.1205727

**Published:** 2013-06-21

**Authors:** Megan N. Hall, Megan Niedzwiecki, Xinhua Liu, Kristin N. Harper, Shafiul Alam, Vesna Slavkovich, Vesna Ilievski, Diane Levy, Abu B. Siddique, Faruque Parvez, Jacob L. Mey, Alexander van Geen, Joseph Graziano, Mary V. Gamble

**Affiliations:** 1 Department of Epidemiology,; 2 Department of Environmental Health Sciences, and; 3 Department of Biostatistics, Mailman School of Public Health, Columbia University, New York, New York, USA; 4 Columbia University Arsenic Project in Bangladesh, Dhaka, Bangladesh; 5 Lamont-Doherty Earth Observatory of Columbia University, Palisades, New York, USA

## Abstract

Background: *In vitro* and rodent studies have shown that arsenic (As) exposure can deplete glutathione (GSH) and induce oxidative stress. GSH is the primary intracellular antioxidant; it donates an electron to reactive oxygen species, thus producing glutathione disulfide (GSSG). Cysteine (Cys) and cystine (CySS) are the predominant thiol/disulfide redox couple found in human plasma. Arsenic, GSH, and Cys are linked in several ways: *a*) GSH is synthesized via the transsulfuration pathway, and Cys is the rate-limiting substrate; *b*) intermediates of the methionine cycle regulate both the transsulfuration pathway and As methylation; *c*) GSH serves as the electron donor for reduction of arsenate to arsenite; and *d*) As has a high affinity for sulfhydryl groups and therefore binds to GSH and Cys.

Objectives: We tested the hypothesis that As exposure is associated with decreases in GSH and Cys and increases in GSSG and CySS (i.e., a more oxidized environment).

Methods: For this cross-sectional study, the Folate and Oxidative Stress Study, we recruited a total of 378 participants from each of five water As concentration categories: < 10 (*n* = 76), 10–100 (*n* = 104), 101–200 (*n* = 86), 201–300 (*n* = 67), and > 300 µg/L (*n* = 45). Concentrations of GSH, GSSG, Cys, and CySS were measured using HPLC.

Results: An interquartile range (IQR) increase in water As was negatively associated with blood GSH (mean change, –25.4 µmol/L; 95% CI: –45.3, –5.31) and plasma CySS (mean change, –3.00 µmol/L; 95% CI: –4.61, –1.40). We observed similar associations with urine and blood As. There were no significant associations between As exposure and blood GSSG or plasma Cys.

Conclusions: The observed associations are consistent with the hypothesis that As may influence concentrations of GSH and other nonprotein sulfhydryls through binding and irreversible loss in bile and/or possibly in urine.

Citation: Hall MN, Niedzwiecki M, Liu X, Harper KN, Alam S, Slavkovich V, Ilievski V, Levy D, Siddique AB, Parvez F, Mey JL, van Geen A, Graziano J, Gamble MV. 2013. Chronic arsenic exposure and blood glutathione and glutathione disulfide concentrations in Bangladeshi adults. Environ Health Perspect 121:1068–1074; http://dx.doi.org/10.1289/ehp.1205727

## Introduction

Arsenic (As) is a class I human carcinogen that causes several types of cancer (International Agency for Research on Cancer 2004). Chronic As exposure through contaminated drinking water has also been linked to increased mortality (Argos et al. 2010) and both coronary (Chen et al. 2011; States et al. 2009) and respiratory diseases (Parvez et al. 2008, 2010). Approximately 140 million people worldwide are chronically exposed to As-contaminated drinking water at concentrations > 10 µg/L; > 57 million of these exposed individuals live in Bangladesh (Kinniburgh and Smedley 2001; World Bank 2005).

**Figure 1:**
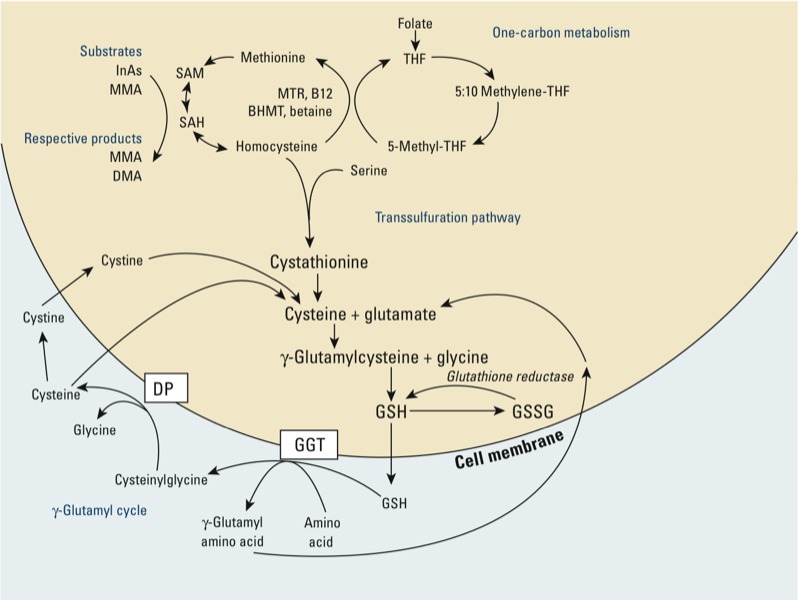
One-carbon metabolism and glutathione (GSH) synthesis and metabolism. Abbreviations: B12, vitamin B_12_; BHMT, betaine homocysteine methyltransferase; DMA, dimethylarsinic acid; InAs, inorganic As; MMA, monomethylarsonic acid. Folic acid is reduced to tetrahydrofolate (THF) and subsequently converted to 5-methyl THF. In a reaction catalyzed by methionine synthetase (MTR), the methyl group of 5‑methyl-THF can be transferred to homocysteine, generating methionine. Methionine is activated to form *S*‑adenosylmethionine (SAM), the universal methyl donor. The by-product of methylation reactions, *S*‑adenosylhomocysteine (SAH), is hydrolyzed to homocysteine. Homocysteine is either used to regenerate methionine or is directed to the transsulfuration pathway. GSH is a product of the transsulfuration pathway. GSH can serve as a continuous source of cysteine, which is extremely unstable, via the γ‑glutamyl cycle (adapted from Lu 2009). GSH is exported from the cell, and the enzyme γ‑glutamyltransferase (GGT) transfers the γ-glutamyl moiety of GSH to an amino acid, often cystine, producing cysteinylglycine and γ-glutamyl amino acid. The γ-glutamyl amino acid can be transported back into the cell and ultimately metabolized to glutamate. Cysteinylglycine is converted to cysteine and glycine by dipeptidase (DP). Cysteine is unstable extracellularly and can oxidize to cystine; both cysteine and cystine can be imported back into the cell for GSH production.

Inorganic As (InAs) undergoes methylation via nutrient-dependent one-carbon metabolism, first to monomethylarsonic acid (MMAs^V^), and then to dimethylarsinic acid (DMAs^V^) (Figure 1). Arsenic metabolites differ in toxicity, and individuals vary in their ability to methylate InAs. Epidemiologic studies have shown that a lower capacity to methylate InAs to DMA^V^, based on relative amounts of As metabolites in urine, is positively associated with risk for several diseases (Steinmaus et al. 2010). The precise mechanisms through which As causes disease are poorly understood, although oxidative stress (defined as an imbalance between antioxidant defense and the total burden of potentially harmful reactive biochemical species) is often cited as a likely mechanism (Jomova et al. 2011).

Glutathione (GSH), a tripeptide consisting of cysteine (Cys), glutamic acid, and glycine, is a critical component of the body’s primary antioxidant defense mechanism (Forman et al. 2009). GSH also serves as the electron donor for the reduction of arsenate (As^V^) to arsenite (As^III^) (Scott et al. 1993). GSH can detoxify reactive oxygen species (ROS) directly and is also a substrate for glutathione peroxidase, a selenoprotein that enzymatically reduces hydrogen peroxide and lipid hydroperoxides (Lu 2009). Detoxification of ROS produces the oxidized metabolite glutathione disulfide (GSSG), which can be reduced back to GSH through the action of glutathione reductase. Another critical function of GSH is to provide a reservoir of Cys in order to maintain overall sulfur amino acid balance (Figure 1). Cells can tolerate only low concentrations of Cys, which is unstable and rapidly oxidizes to cystine (CySS), producing potentially toxic oxygen free radicals (Lu 2009).

A decrease in GSH and increase in GSSG is indicative of chronic oxidative stress, and severe oxidative stress has been shown to deplete GSH (Lu 1999). Furthermore, changes in the reduction potential (E_h_) of the GSH/GSSG and Cys/CySS thiol/disulfide pairs may influence redox-sensitive signaling pathways and enzymes (Jones 2002). Concentrations of reduced and oxidized forms of a paired redox couple can be used to estimate the redox state (in millivolts) with the Nernst equation (Jones et al. 2002). The GSH/GSSG thiol/disulfide redox couple maintains a highly reduced intracellular state that controls cell cycle progression and intracellular antioxidant defenses (Schafer and Buettner 2001). In plasma, Cys and CySS are present at much higher concentrations than GSH and GSSG, and are thus the primary determinants of the extracellular redox state (Jones et al. 2002). The high intracellular (1–10 mM) and lower extracellular (1–10 µM) concentrations of GSH might render extracellular surfaces more vulnerable to oxidative damage (Lu 2009; Smith et al. 1996); this may be partially counteracted by the high extracellular concentrations of the Cys/CySS redox pair.

One-carbon metabolism is the biochemical pathway through which As and numerous other substrates are methylated (Figure 1). A portion of homocysteine generated through one-carbon metabolism is remethylated to form methionine, which is then activated to form the methyl donor, *S*-adenosyl methionine (SAM). Alternatively, homocysteine can be directed to the transsulfuration pathway and used in the synthesis of GSH (Figure 1). Under conditions of oxidative stress, the shunting of homocysteine to the transsulfuration pathway is increased through the up-regulation of the GSH biosynthetic enzymes, including γ-glutamate-cysteine ligase (GCL), the rate-limiting enzyme of GSH synthesis (Lu 2009). Although most cell types can synthesize GSH, the liver is the major site of synthesis.

There are several mechanisms through which As may influence concentrations of GSH and GSSG, including *a*) inducing the formation of ROS, which are then detoxified by GSH; *b*) consuming GSH during the reduction of As^V^ to As^III^ (Scott et al. 1993); *c*) forming As/GSH complexes, which are substrates for the ATP-binding cassette membrane transporters that mediate efflux from cells (Leslie et al. 2004; Thomas 2009); and *d*) inhibiting glutathione reductase, thereby limiting the regeneration of GSH from GSSG (Massey and Williams 1965; Styblo et al. 1997; Styblo and Thomas 1995). *In vitro* and animal studies have shown that As can deplete GSH (Santra et al. 2000). Thus, the depletion of GSH may be one mechanism through which As could lead to oxidative stress. However, given the well-known species differences in As metabolism and susceptibility to As-induced health effects (Drobná et al. 2010), additional data derived from human population studies are needed.

We conducted a cross-sectional study of 378 Bangladeshi adults to test the primary hypothesis that chronic As exposure is associated with reductions in GSH and increases in GSSG. We also wished to test the secondary hypothesis that chronic As exposure is associated with reductions in plasma Cys and increases in CySS.

## Subjects and Methods

*Participants and procedures.* Participants in the current study, the Folate and Oxidative Stress (FOX) Study, were recruited between February and July of 2008 in Araihazar, Bangladesh. Potential participants were identified on the basis of well water As (wAs) concentrations obtained from a well survey in the year 2000 (van Geen et al. 2002) in order to ensure a wide range of As exposures for the examination of dose-dependent relationships. A new water sample was collected at the time of enrollment for analysis of wAs concentration. Individuals were eligible to participate in FOX if they *a*) were between 30 and 65 years of age; *b*) were not pregnant; *c*) were not taking nutritional supplements; *d*) did not have known diabetes, cardiovascular or renal disease, chronic obstructive pulmonary disease, or cancer; and *e*) had been drinking water from their current well for at least 3 months. Trained recruiters identified eligible participants, explained the nature of the study, obtained informed consent, and scheduled a field clinic visit. Because GSH is unstable and therefore requires that blood samples be processed immediately, all visits were conducted in the laboratory at our field clinic in Araihazar. During the field clinic visit, a trained interviewer administered a detailed questionnaire to each participant, and a physician collected a venous blood sample. Urine samples were collected in 50-mL acid-washed polypropylene tubes and frozen at –20°C.

The primary aim of this study was to examine the dose–response relationship between As exposure and measures of oxidative stress. We aimed to recruit 75 participants each from five wAs concentration categories: < 10, 10–100, 101–200, 201–300, and > 300 µg/L. However, the final sample included more particpants with lower exposures because many households switched to lower-As wells after wells in the region were surveyed for As in 1999–2000 (Chen et al. 2007). Therefore, the final distribution among well wAs exposure categories was < 10 µg/L (*n* = 76), 10–100 µg/L (*n* = 104), 101–200 µg/L (*n* = 86), 201–300 µg/L (*n* = 67), and > 300 µg/L (*n* = 45).

Oral informed consent was obtained by our Bangladeshi field staff physicians, who read an approved assent form to the study participants. This study was approved by the Bangladesh Medical Research Council and the institutional review board of Columbia University Medical Center.

*Sample collection and handling.* After the initial processing of blood samples in the field clinic, the blood and plasma aliquots were immediately frozen at –80°C. Samples on dry ice were transported in batches to Dhaka, Bangladesh, by car and again stored at –80°C (blood and plasma) or –20°C (urine). Samples were then packed on dry ice in coolers and transported by air to Columbia University.

*wAs.* Field sample collection and laboratory analysis procedures have been previously described in detail (Cheng et al. 2004; van Geen et al. 2005). Water samples were collected in 20-mL polyethylene scintillation vials. The samples were acidified to 1% with high-purity Optima HCl (Fisher Scientific, Pittsburg, PA, USA) at least 48 hr before analysis (van Geen et al. 2007). Water samples were analyzed by high-resolution inductively coupled plasma mass spectrometry (ICP-MS) after 1:10 dilution and addition of a germanium spike to correct fluctuations in instrument sensitivity. The detection limit of the method is typically < 0.2 μg/L (Cheng et al. 2004). A standard with an As concentration of 51 µg/L was run multiple times in each batch. The intraassay and interassay coefficients of variation (CVs) for this standard were 6.01% and 3.76%, respectively.

*Total urinary As.* All urine samples were analyzed for total urinary As (uAs) in the Columbia University Trace Metals Core Laboratory by graphite furnace atomic absorption spectrometry (Nixon et al. 1991) using the AAnalyst 600 graphite furnace system (PerkinElmer, Shelton, CT, USA). A method based on the Jaffe reaction was used to measure urinary creatinine concentrations (Slot 1965). Method and instrument precision was checked by running four different urine samples with known concentrations (to cover the whole linearity range of the standard curve) every day immediately after the instrument calibration with aqueous standards. A urine sample with an As concentration in the middle of the linearity range was run after every 10 study samples. The intraassay and interassay CVs based on this quality control sample were 3.9% and 5.6%, respectively. For duplicate study samples, the intraassay and interassay CVs were 3.8% and 5.1%, respectively.

*Total blood As.* We used a Perkin-Elmer Elan DRC II ICP-MS equipped with an AS 93+ autosampler to analyze whole blood samples for total blood As (bAs) concentration, as described previously (Hall et al. 2006). The intraassay and interassay CVs were 3.2% and 5.7%, respectively, for quality control samples. For study samples, the intraassay and interassay CVs were 2.1% and 4.9%, respectively.

*Blood GSH and GSSG and plasma Cys and CySS.* Whole blood GSH and GSSG and plasma Cys and CySS were assayed essentially as described by Jones et al. (1998). Blood was collected with a butterfly needle and syringe and then immediately transferred into Eppendorf tubes. For whole blood measurements, the Eppendorf tubes contained 5% perchloric acid, 0.1 M boric acid, and γ-glutamyl glutamate as an internal standard. For plasma measurements, the tubes contained 0.53 g l-serine, 25 mg heparin, 50 mg bathophenanthrolene, 300 mg iodoacetic acid, and 10 mL borate buffer stock (12.4 g boric acid, 19 g sodium tetraborate decahydrate, and 500 mL distilled water). The samples for plasma measurements were centrifuged for 1 min, and 200 µL of supernatant was transferred into Eppendorf tubes containing an equal volume of 10% perchloric acid and 0.2 M boric acid. For derivatization, plasma samples were centrifuged at 13,000 rpm for 2 min, 300 µL of supernatant was transferred to a fresh tube, and the pH was adjusted to 9.0. After incubating for 20 min at room temperature, dansyl chloride was added, and samples were incubated at room temperature in the dark for 24 hr. The derivatized samples were then stored at –80°C until delivered to Columbia University for analysis. Free dansyl chloride was extracted from thawed samples with 500 µL chloroform, and then 20 µL of the sample was injected onto the HPLC. Separation was achieved using a Supelcosil LC-NH_2_ column (catalog no. 58338; Sigma Chemical Co., St. Louis, MO, USA). Initial solvent conditions were 60% A (80% methanol, 20% water), 40% B (acetate-buffered methanol, pH 4.6) run at 1 mL/min for 10 min. A linear gradient to 20% A, 80% B was run during the 10- to 50-min period. From 50 to 52 min, the conditions were returned to 60% A, 40% B. Metabolites were detected using a Waters 474 scanning fluorescence detector (Waters Corp., Milford, MA, USA), with 335 nm excitation and 515 nm emission. Within-assay CVs were all between 0.05 and 0.10, and interassay CVs were between 0.11 and 0.18.

*Plasma folate.* Plasma folate was analyzed by radio protein-binding assay (SimulTRAC-S; MP Biomedicals, Orangeburg, NY, USA). To determine folate concentrations, we used folic acid as pteroylglutamic acid for calibration, and its ^125^I-labeled analog as the tracer. The intraassay and interassay CVs were 0.06 and 0.14, respectively.

*Calculation of the reduction potential.* The reduction potential (E_h_) of the thiol/disulfide GSH/GSSG and Cys/CySS redox pairs (blood GSH E_h_ and plasma Cys E_h_, respectively) were calculated using the Nernst equation:

*E*_h_ = *E*_o_ + *RT*/*nF* ln(acceptor/(donor)^2^,

where *E*_o_ is the standard potential for the redox couple, *R* is the gas constant, *T* is the absolute temperature, *n* = 2 for the number of electrons transferred, and *F* is Faraday’s constant (Jones et al. 2002). For GSH and GSSG, the equation simplifies to

*E*_h_ (mV) = −264 + 30 log[(GSSG)/(GSH^2^)],

where (GSH) and (GSSG) are molar concentrations, and the *E*_o_ value assumes a physiologic pH of 7.4. A more positive *E*_h_ value indicates a more oxidized redox state.

*Statistical methods.* We calculated descriptive statistics for characteristics of the study sample, As exposure variables (wAs, uAs, and bAs), and outcome variables (blood GSH and GSSG, plasma Cys and CySS), both for the total sample and by sex. Bivariate associations were examined using scatter plots and Spearman’s correlation coefficients. To examine the bivariate associations between dichotomous covariates and As exposure variables or continuous outcome variables we used *t*-tests or the nonparametric Wilcoxon rank sum test.

We used linear regression models to further examine the associations between As exposure variables, as continuous variables, and the outcome variables, with and without adjustment for potential confounders. Age and sex were included in all covariate-adjusted regression models. Other covariates considered for inclusion in the regression models were variables reported to be associated with the exposures or outcomes based on previous publications and/or variables associated with the exposure and outcome variables in the present study population. These variables included television ownership (as a surrogate for socioeconomic status), cigarette smoking, body mass index (BMI), urinary creatinine, and plasma folate. We adjusted for GSH laboratory batch (as a categorical variable) in order to reduce extraneous variation in the outcome variables. We also calculated the change in *R*^2^ between models for each outcome that included covariates only and corresponding models that included both the covariates and As exposure.

To facilitate comparisons among the different measures of exposure (wAs, uAs, and bAs), we report the estimated change in the mean value of blood GSH, blood GSH E_h_, plasma CySS, and plasma Cys E_h_ associated with an interquartile range (IQR) increase in each exposure. For outcome variables that were natural log-transformed (blood GSSG and plasma Cys) we report the ratio of estimated geometric means for an IQR change in As exposure.

To examine possible nonlinear relationships, we also created quintiles of As exposure variables and computed covariate-adjusted mean values of the outcome variables for categories of As exposure; plots of quintile-specific adjusted mean values were examined to determine if the association was approximately linear.

We ran separate linear regression models to examine the covariate-adjusted associations between As exposure and the outcome variables stratified by sex or by folate status. We then used a Wald test to detect differences in the covariate-adjusted associations between As exposure and outcome variables by sex or by folate status. All analyses were performed using SAS (version 9.2; SAS Institute Inc., Cary, NC, USA); all statistical tests were two-sided with a significance level of 0.05.

## Results

The general characteristics of the study sample are shown in Table 1. The median age was 42 years, approximately half of the study participants were female, and the median BMI was 19.7. wAs exposure ranged from 0.4 to 700 µg/L; by design, nearly 80% of the participants had wAs concentrations above the World Health Organization (WHO) standard of 10 µg/L for drinking water (WHO 2004). As we have observed previously (Gamble et al. 2005), males and females differed on the following characteristics: Males *a*) had more years of formal education (median: males, 4; females, 1; *p* = 0.001); *b*) were more likely to report being ever-smokers (males, 69%; females, 5.7%; *p* < 0.0001); *c*) were more likely to have a BMI < 18.5 (males, 40.8%; females, 26.4%; *p* = 0.003); *d*) were more likely to have plasma folate concentrations < 9 nmol/L (males, 38.8%; females, 20.6%; *p* < 0.0001), which is indicative of marginal folate status; and *e*) were more likely to have total homocysteine concentrations above the cutoff for hyperhomocysteinemia of 13 µmol/L (males, 25.5%; females, 7.2%; *p* = 0.0001). We observed other differences between the sexes that were specific to the present study sample: The males were older than the females (median: males, 44.0 years; females, 40.0 years; *p* = 0.001) and had higher bAs concentrations (median: males: 12.3 µg/L; females, 10.3 µg/L; *p* = 0.009). Blood GSH E_h_ and plasma Cys E_h_ also differed significantly by sex; median blood GSH E_h_ values for females indicated a more oxidized intracellular redox state compared with males (median: males, –203.2; females, –197.4; *p* < 0.0001), whereas median plasma Cys E_h_ values for males indicated a more oxidized extracellular redox state compared with females (median: males, –46.1; females, –49.4; *p* = 0.03).

**Table 1:** Characteristics of the study sample, for all participants and by sex.^*a*^

Characteristic	Total sample (*n *= 378)	Females (*n *= 194)	Males (*n *= 184)
Age (years)	42.0 (13.0)	40.0 (13.0)	44.0 (14.0)
Education (years)	3.0 (5.0)	1.0 (5.0)	4.0 (6.0)
BMI (kg/m^2^)	19.7 (5.0)	20.7 (5.0)	19.0 (4.2)
BMI < 18.5 (%)	33.4	26.4	40.8
Ever-smoker (%)	36.5	5.7	69.0
Ever betel nut use (%)	42.6	41.2	44.0
Television ownership (%)	58.2	57.2	59.2
wAs (μg/L)	114.0 (190.3)	113.1 (186.9)	114.1 (215.2)
wAs > 10 μg/L (%)	79.9	80.4	79.4
wAs > 50 μg/L (%)	68.3	68.6	67.9
uAs (μg/L)	122.5 (223.0)	119.0 (217.0)	126.0 (218.0)
Urinary creatinine (mg/dL)	40.3 (57.0)	36.2 (49.8)	44.0 (57.1)
uAs/g creatinine	329.5 (454.0)	360.0 (465.0)	307.5 (392.0)
bAs (μg/L)	10.9 (13.3)	10.3 (12.4)	12.3 (14.3)
Plasma folate (nmol/L)	11.1 (6.5)	12.5 (6.9)	9.7 (5.1)
Folate deficient, < 9 nmol/L (%)	29.4	20.6	38.8
Homocysteine (μmol/L)	9.0 (4.3)	7.7 (3.5)	10.7 (5.1)
Hyperhomocysteinemia, > 13 μmol/L (%)	16.1	7.2	25.5
Blood GSH (μmol/L)	494.6 (227.3)	436.6 (212.0)	529.7 (209.7)
Blood GSSG (μmol/L)	34.3 (23.4)	34.5 (20.6)	33.9 (25.8)
Blood GSH E_h_	–199.9 (14.3)	–197.4 (12.4)	–203.2 (12.3)
Plasma Cys (μmol/L)	3.2 (2.8)	3.5 (2.5)	2.9 (2.9)
Plasma CySS (μmol/L)	55.5 (19.0)	53.6 (19.7)	56.2 (19.0)
Plasma Cys E_h_	–48.5 (22.6)	–49.4 (19.3)	–46.1 (24.0)
Abbreviations: bAs, blood As; Cys, cysteine; CySS, cystine; E_h_, reduction potential; GSH, glutathione; GSSG, glutathione disulfide; uAs, urinary As; wAs, water As. ^***a***^Values are median (interquartile range) unless otherwise noted.			

Several covariates were associated with As exposure variables and/or outcome variables. All three measures of As exposure were negatively associated with BMI (see Supplemental Material, Table S1). Plasma folate was positively associated with blood GSSG. Age, BMI, and plasma folate were all positively associated with plasma CySS; age was also negatively associated with plasma Cys. We also observed differences by sex, smoking, and television ownership (see Supplemental Material, Table S2). Males had higher blood GSH and lower plasma Cys and blood GSH E_h_ (indicating a less oxidized redox state) than did females. Ever-smokers also had significantly higher bAs than never-smokers. Contrary to expectation, ever-smokers had higher blood GSH concentrations and lower blood GSH E_h_ than never-smokers. However, most smokers were males, and after stratification by sex, there were no statistically significant difference in blood GSH and blood GSH E_h_ by smoking status (data not shown). Participants who owned a television had lower wAs and uAs concentrations and higher blood GSH, blood GSSG, and plasma CySS concentrations than participants who did not own a television.

In the models in which we computed covariate-adjusted mean values of the outcome variables for each quintile of As exposure, we did not detect any substantial departures from linearity (data not shown). In models using continuous As exposure variables, all three markers of As exposure were negatively associated with blood GSH [estimated changes in mean blood GSH associated with an IQR increase in exposure from covariate-adjusted models were –25.4 µmol/L (95% CI: –45.3, –5.31), *p* = 0.01 for wAs; –54.0 µmol/L (95% CI: –90.8, –17.2), *p* = 0.004 for uAs; and –33.4 µmol/L (95% CI: –62.8, –3.9), *p* = 0.03 for bAs] and positively—but not always significantly—associated with blood GSH E_h_ [change in the estimated mean with an IQR increase in exposure from covariate-adjusted models: 1.19 (95% CI: –0.25, 2.62), *p* = 0.10 for wAs; 2.72 (95% CI: 0.10, 5.35), *p* = 0.04 for uAs, and 1.73 (95% CI: –0.37, 3.83), *p* = 0.11 for bAs] (Table 2). The change in *R*^2^ values for blood GSH models were 1.3% for wAs, 1.8% for uAs, and 1.1% for bAs; these values can be interpreted as the proportion of the variance in blood GSH that is accounted for by each As exposure variable after controlling for covariates. We observed no significant associations between As exposure and blood GSSG concentrations (Table 2). In unadjusted models, wAs and bAs were significantly negatively associated with plasma Cys. However, associations were positive (although not statistically significant) after adjustment for covariates. All three markers of As exposure were negatively associated with plasma CySS in both covariate-unadjusted and covariate-adjusted models [estimated changes in mean plasma CySS associated with an IQR increase in exposure from covariate-adjusted models were –3.00 µmol/L (95% CI: –4.61, –1.40; *p* = 0.0002) for wAs; –3.56 µmol/L (95% CI: –6.47, –0.66; *p* = 0.02) for uAs, and –3.09 µmol/L (95% CI: –5.40, –0.79; *p* = 0.009) for bAs] and negatively associated with plasma Cys E_h_ [estimated changes in mean plasma Cys E_h_ associated with an IQR increase in exposure from covariate-adjusted models were –2.05 µmol/L (95% CI: –3.89, –0.21; *p* = 0.03) for wAs; –4.04 µmol/L (95% CI: –7.34, –0.74; *p* = 0.02) for uAs; and –2.01 µmol/L (95% CI: –4.65, 0.64; *p* = 0.14) for bAs], indicating that As exposure is associated with a more reduced extracellular environment. The change in *R*^2^ values for plasma CySS models with adjustment for As exposure (compared with covariates alone) were 2.7% for wAs, 1.2% for uAs, and 1.4% for bAs. Given that males and females in this study population differed substantially on several characteristics, all regression models were also run stratified by sex; the directions of associations in males and females were the same as in the overall sample, but in some cases did not reach statistical significance because of the smaller sample sizes (data not shown). We also examined the possibility that associations might differ by folate status (deficient: < 9 nmol/L, sufficient: ≥ 9 nmol/L) given the influence of folate on As methylation (Gamble et al. 2006). We observed no statistically significant differences in the associations between blood As and any of the outcome variables by folate status (see Supplemental Material, Table S3).

**Table 2:** Covariate-unadjusted and -adjusted effect size estimates for associations between measures of As exposure and GSH, GSSG, Cys, and CySS.

	wAs (μg/L)		uAs (μg/L)		bAs (μg/L)	
	Unadjusted	Adjusted^*a*^	Unadjusted	Adjusted^*b*^	Unadjusted	Adjusted^*a*^
Blood GSH (μmol/L)
Mean change (95% CI)^*c*^	–33.8 (–49.3, –18.0)	–25.4 (–45,3, –5.31)	–59.6 (–84.0, –35.3)	–54.0 (–90.8, –17.2)	–46.6 (–72.3, –21.1)	–33.4 (–62.8, –3.9)
*p*-Value	< 0.0001	0.01	< 0.0001	0.004	0.0004	0.03
*R*^2^	4.6	23.5	5.8	24.8	3.3	23.3
∆*R*^2^		1.3		1.8		1.1
Blood GSSG (μmol/L)
Mean ratio (95% CI)^*d*^	1.00 (0.95, 1.04)	0.98 (0.95, 1.02)	1.07 (0.99, 1.14)	0.95 (0.88, 1.05)	0.99 (0.92, 1.06)	0.98 (0.91, 1.04)
*p*-Value	0.88	0.42	0.07	0.31	0.63	0.43
*R*^2^	0.0	47.8	0.8	48.0	0.06	47.8
∆*R*^2^		0.1		0.2		0.1
Blood GSH E_h_ (mV)^*e*^
Mean change (95% CI)^*c*^	1.92 (0.74, 3.11)	1.19 (–0.25, 2.62)	4.51 (2.67, 6.35)	2.72 (0.10, 5.35)	2.64 (0.70, 4.58)	1.73 (–0.37, 3.83)
*p*-Value	0.002	0.10	< 0.0001	0.04	0.008	0.11
*R*^2^	2.6	31.1	5.8	32.2	1.9	31.1
∆*R*^2^		0.5		0.8		0.5
Plasma Cys (μmol/L)
Mean ratio (95% CI)^*d*^	0.93 (0.88, 0.98)	1.06 (0.98, 1.14)	0.98 (0.89, 1.09)	1.12 (0.99, 1.28)	0.90 (0.81, 0.99)	1.05 (0.95, 1.16)
*p*-Value	0.01	0.14	0.73	0.06	0.03	0.34
*R*^2^	1.7	39.4	0.0	40.6	1.3	39.2
∆*R*^2^		0.3		0.6		0.1
Plasma CySS (μmol/L)
Mean change (95% CI)^*c*^	–2.94 (–4.19, –1.69)	–3.00 (–4.61, –1.40)	–1.78 (–3.80, 0.25)	–3.56 (–6.47, –0.66)	–4.11 (–6.17, –2.06)	–3.09 (–5.40, –0.79)
*p*-Value	< 0.0001	0.0002	0.08	0.02	0.0001	0.009
*R*^2^	5.3	29.8	0.8	28.8	4.0	28.5
∆*R*^2^		2.7		1.2		1.4
Plasma Cys E_h_ (mV)^*f*^
Mean change (95% CI)^*c*^	1.38 (–0.21,2.98)	–2.05 (–3.89, –0.21)	–0.007 (–2.51, 2.49)	–4.04 (–7.34, –0.74)	1.90 (–0.67, 4.48)	–2.01 (–4.65, 0.64)
*p*-Value	0.09	0.03	0.99	0.02	0.15	0.14
*R*^2^	0.8	38.3	0.0	39.1	0.6	37.8
∆*R*^2^		0.9		1.0		0.4
Abbreviations: bAs, blood As; Cys, cysteine; CySS, cystine; E_h_, reduction potential; GSH, glutathione; GSSG, glutathione disulfide; uAs, urinary As; wAs, water As. ^***a***^Adjusted for log-transformed age, sex, television ownership (yes/no), smoking (ever/never), log-transformed BMI and plasma folate, and GSH/Cys laboratory batch. ­ ^***b***^Also adjusted for log urinary creatinine. ^***c***^Represents the mean change in the outcome for a change in the exposure from the 25th to the 75th percentile. ^***d***^Represents the ratio of the geometric mean in the outcome for a change in the exposure from the 25th to the 75th percentile. ^***e***^Reduction potential of the GSH/GSSG redox pair. ^***f***^Reduction potential of the Cys/CySS redox pair.						

## Discussion

In this cross-sectional study of Bangladeshi adults chronically exposed to a wide range of As concentrations in drinking water, we observed that three different measures of As exposure (wAs, uAs, and bAs) were all negatively associated with blood GSH. Contrary to our primary *a priori* hypothesis, As exposure was not positively associated with blood GSSG, the oxidized form of GSH. Also, contrary to our secondary hypotheses, we observed no association between As and plasma Cys, and we saw a negative—rather than positive—association with plasma CySS.

To our knowledge, only one other population-based study has examined the association between As exposure and GSH concentrations. Xu et al. (2008) recruited 208 participants with high wAs exposure (90 µg/L or 160 µg/L) and 59 participants with low wAs exposure (20 µg/L) from three villages in Inner Mongolia, China. Participants with high wAs exposure had significantly lower blood total non-protein sulfhydryl concentrations (NPSH, primarily GSH) than those with low wAs exposure. Xu et al. (2008) did not report findings for blood GSSG.

Our findings and those of Xu et al. (2008) are generally consistent with rodent studies showing that high-dose As exposure depletes GSH (Maiti and Chatterjee 2001; Santra et al. 2000). Santra et al. (2000) reported that mice administered As through drinking water (3,200 µg/L) *ad libitum* had elevated hepatic GSH after 2 months and decreased hepatic GSH after 4 months compared with mice administered As-free water. In male Wistar rats, Maiti and Chatterjee (2001) found that a single intraperitoneal dose of sodium arsenite (15,860 µg/kg body weight) resulted in a significant reduction in hepatic GSH.

Despite the evidence suggesting that As exposure is associated with reductions in GSH, the question of whether or not chronic exposure—at doses relevant to human populations—results in oxidative stress per se remains unclear. Some *in vitro* and animal model studies have reported that As exposure led to an increase in the production of ROS and a decline in GSH (Flora et al. 2012; Thompson and Franklin 2010; Zhang et al. 2011). However, these studies used high doses of As, doses that are often not relevant to actual human exposures. For example, Flora et al. (2012) administered an As dose of 50 ppm in drinking water (~ 50,000 µg/L) to mice. We would not expect very high doses to produce the same effects as the doses to which our study participants were exposed (median, 114 µg/L). In addition, there are well known species differences in both As metabolism and susceptibility to As-induced health effects (Drobná et al. 2010).

The negative associations between As exposure and blood GSH and plasma CySS, although statistically significant, accounted for a small proportion of the estimated variance in blood GSH (1.1–1.8%) and plasma CySS (1.2–2.7%). Our findings, as well as the inconsistent findings from other population-based studies examining associations between As exposure and blood markers of oxidative damage (Burgess et al. 2007; Engström et al. 2010a, 2010b; Pi et al. 2002), raise the possibility that, despite reductions in GSH, As may not induce substantial, sustained oxidative stress that is measurable in blood using these biomarkers at environmentally relevant As exposures. We cannot determine from this study what the functional effects of the observed reduction in blood GSH might be; however, alterations in GSH have been linked to the development and/or progression of numerous diseases (Ballatori et al. 2009).

Research by Waalkes and colleagues suggests that human cells can adapt to low-level As exposure through alterations in the one-carbon metabolic pathway (Coppin et al. 2008). Exposure of human prostate epithelial RWPE-1 cells (which do not methylate As) to 5 µM sodium arsenite for up to 16 weeks resulted in reductions in SAM and increases in *S*-adenosylhomocysteine and homocysteine. In addition, As exposure increased the expression of cystathionine β-synthase, the enzyme that catalyzes the first step in GSH production via the transsulfuration pathway, and increased cellular reduced GSH by 5-fold (Coppin et al. 2008). Collectively, these findings suggest that in RWPE-1 cells, low-level As exposure increases shunting of homocysteine to the transsulfuration pathway to increase GSH production, resulting in decreased remethylation of homocysteine to SAM. Exposure to As also resulted in increased expression of the ATP-binding cassette protein C1 (ABBC1) and increased efflux of As from RWPE-1cells (Coppin et al. 2008). Given that *ABBC1* encodes a protein involved in the efflux of a triglutathione-arsenical complex (Leslie et al. 2004), the findings of Coppin et al. (2008) suggest that As may exert a strong influence on GSH concentrations through this mechanism.

We observed a negative association between As exposure and blood GSH but no association with blood GSSG, a finding that is most consistent with the hypothesis that As may reduce GSH through binding and irreversible loss in bile and/or possibly in urine (Kala et al. 2004). This finding also suggests that other proposed As-mediated mechanisms [specifically, GSH depletion during the detoxification of ROS; GSH depletion during the reduction of As^V^ to As^III^ (Scott et al. 1993); or As-mediated glutathione reductase inhibition resulting in reduced regeneration of GSH from GSSG (Styblo et al. 1997)] did not strongly influence GSH status in our chronically exposed Bangladeshi study population. Associations between the IQR increases in wAs and bAs and the outcomes were generally quite similar (Table 2). For blood GSH, blood GSH E_h_, plasma CySS, and plasma Cys E_h_, the magnitudes of the associations were somewhat stronger for an IQR increase in uAs compared with IQR increases in wAs and bAs.

A secondary hypothesis of the present study was that As would be negatively associated with Cys because Cys is required for GSH synthesis. However, our findings showed a positive but not statistically significant association with plasma Cys and a negative association with CySS. The lack of a statistically significant association with Cys may potentially be explained by the fact that free Cys is extremely unstable extracellularly and rapidly oxidizes to CySS (Lu 2009). In fact, one of the functions of GSH is to serve as a storage form of Cys through the γ-glutamyl cycle (Meister and Anderson 1983). Given that Cys is constantly in flux, the measurement of plasma Cys may reflect one snapshot of Cys concentrations rather than the overall Cys status of an individual. In a study designed to examine the long-term stability of blood glutathione and cyst(e)ine (i.e., cysteine + cystine) measurements, Richie et al. (1996) reported low interindividual variability in cyst(e)ine, which suggests that measures of Cys and/or CySS in whole blood or plasma may not be as useful as a biomarker of long-term cyst(e)ine status.

A number of factors influence GSH concentrations (including age, smoking, physical activity, and diet), and an individual’s response to As might depend on their background level of oxidative stress. A mathematical model of one-carbon metabolism developed by Reed et al. (2008) predicted that changes in GSH and Cys with oxidative stress depend on the severity of the oxidative stress. For example, with moderate overall oxidative stress, blood and cytosolic GSH and cytosolic Cys increase while blood Cys decreases. This initial increase in GSH results from the stimulation of enzymes involved in GSH synthesis. With severe oxidative stress, values of all four metabolites decrease sharply (Reed et al. 2008). In our statistical analysis, we did not find evidence of nonlinear relationships between As exposure and the outcome variables (data not shown). In addition, we controlled for several potential confounding factors. However, we were unable to adjust for physical activity, a variable previously shown to be associated with GSH (Rundle et al. 2005), and uncontrolled confounding by this or other unmeasured factors is possible.

## Conclusions

We observed that As exposure was negatively associated with blood GSH and plasma CySS in a population chronically exposed to a wide range of As concentrations in drinking water. These findings, and the lack of association between As exposure and blood GSSG or plasma Cys, suggest that As may exert its greatest influence on GSH through binding and irreversible loss in bile and/or possibly in urine. Our findings do not lend strong support to the hypothesis that As induces levels of oxidative stress that are measurable in blood using these biomarkers and at these environmentally relevant exposures. However, our findings cannot rule out the possibility of As-induced oxidative stress in tissue or cellular compartments that we were unable to measure in healthy human volunteers.

## Correction

In the manuscript originally published online, Abu B. Siddique’s name was listed incorrectly. It has been corrected here.

## Supplemental Material

(532 KB) PDF
