# Supplementary material for: Chronic Arsenic Exposure and Blood Glutathione and Glutathione Disulfide Concentrations in Bangladeshi Adults
Source: Environ Health Perspect. 2013 Jun 21;121(9):1068–74. doi: 10.1289/ehp.1205727 (PMC3764071; doi:10.1289/ehp.1205727)
Supplement: (532 KB) PDF [file ehp.1205727.s001.pdf]

Supplemental Material

Chronic Arsenic Exposure and Blood Glutathione and Glutathione Disulfide Concentrations in Bangladeshi Adults

Megan N. Hall, Megan Niedzwiecki, Xinhua Liu, Kristin N. Harper, Shafiul Alam, Vesna Slavkovich, Vesna Ilievski, Diane Levy, Suman Siddique, Faruque Parvez, Jacob L. Mey, Alexander van Geen, Joseph Graziano, and Mary V. Gamble

Table of Contents

Table S1. Associations of continuous covariates with As exposure and outcome variables…p2

Table S2. Associations of categorical covariates with As exposure and outcome variables...p3

Table S3. Covariate adjusted effect size estimates for associations between measures of As exposure and GSH, GSSG, Cys, and CySS by folate status…...………………….…………p4

Supplemental Material, Table S1. Associations of continuous covariates with As exposure and outcome variables

	Age	BMI	Plasma Folate
Water As	-0.02 (0.73)[a]	-0.12 (0.02)	-0.07 (0.15)
Urinary As	-0.04 (0.46)	-0.11 (0.03)	0.0007 (0.99)
Blood As	0.0005 (0.99)	-0.16 (0.002)	-0.10 (0.06)
Blood GSH	-0.06 (0.25)	0.01 (0.86)	-0.07 (0.17)
Blood GSSG	-0.06 (0.24)	0.05 (0.36)	0.16 (0.002)
Blood GSH Eh	0.009 (0.86)	0.03 (0.55)	0.15 (0.003)
Plasma Cys	-0.13 (0.01)	-0.0005 (0.99)	0.003 (0.95)
Plasma CySS	0.17 (0.001)	0.17 (0.0007)	0.11 (0.03)
Plasma Cys Eh	0.17 (0.001)	0.02 (0.66)	0.02 (0.71)

a – Spearman's correlation coefficients (p-value)

Supplemental Material, Table S2. Associations of categorical covariates with As exposure and outcome variables

	Sex			Ever smoke			TV Ownership		
	**Male[a]**	**Female[a]**	**p-value[b]**	**Yes[a]**	**No[a]**	**p-value[b]**	**Yes[a]**	**No[a]**	**p-value[b]**
Water As	114.1	113.1	0.75	119.4	107.4	0.38	101.2	138.5	0.03
Urinary As/g Cr	307.5	360.0	0.49	340.5	328.5	0.44	306.5	370.5	0.01
Blood As	12.3	10.3	0.009	12.4	10.3	0.008	10.4	11.1	0.17
Blood GSH	529.7	436.6	<0.0001	516.9	457.1	0.004	511.3	462.0	0.08
Blood GSSG	33.9	34.5	0.26	34.1	34.3	0.70	37.4	29.6	<0.0001
Blood GSH Eh	-203.2	-197.4	<0.0001	-202.9	-198.6	0.006	-200.1	-199.5	0.75
Plasma Cys	2.9	3.5	0.04	3.1	3.3	0.45	3.2	3.1	0.97
Plasma CySS	56.2	53.6	0.51	55.6	55.1	0.99	57.5	52.7	0.005
Plasma Cys Eh	-46.1	-49.4	0.03	-46.1	-48.6	0.43	-47.2	-49.0	0.56

a - median values of exposure or outcome variable for covariate category, b – p-value from Wilcoxon rank-sum test

Supplemental Material, Table S3. Covariate adjusted effect size estimates for associations between measures of As exposure and GSH, GSSG, Cys, and CySS by folate status

	Water As (µg/L)[a]		Urinary As (µg/L)[b]		Blood As (µg/L)[a]	
	Folate Sufficient[c]	Folate Deficient[d]	Folate Sufficient[c]	Folate Deficient[d]	Folate Sufficient[c]	Folate Deficient[d]
**Blood GSH (µmol/L)**						
Mean change (95% CI)[e]	-44.6 (-69.7,-19.4)	-10.2 (-41.9,21.5)	-79.4 (-125.1,-38.7)	-39.4 (-97.2,18.5)	-37.3 (-75.2,0.54)	-23.5 (-67.8,20.7)
p-value	0.0006	0.52	0.0007	0.18	0.05	0.29
**Blood GSSG (µmol/L)**						
Mean ratio (95% CI)[f]	1.03 (0.97,1.09)	0.93 (0.84,1.00)	1.01 (0.92,1.12)	0.87 (0.74,1.02)	1.01 (0.93,1.10)	0.90 (0.79,1.01)
p-value	0.34	0.06	0.78	0.08	0.83	0.08
**Blood GSH Eh (mV)** [g]						
Mean change (95% CI)[e]	2.64 (0.85,4.42)	-0.44 (-2.50,1.65)	4.24 (1.01,7.47)	0.44 (-3.35,4.21)	2.75 (0.11,5.40)	0.03 (-2.87,2.92)
p-value	0.004	0.68	0.01	0.82	0.04	0.99
**Plasma Cys (µmol/L)**						
Mean ratio (95% CI)[f]	1.07 (0.99,1.16)	1.03 (0.88,1.20)	1.13 (0.98,1.30)	1.18 (0.89,1.58)	1.04 (0.92,1.16)	1.06 (0.85,1.33)
p-value	0.09	0.71	0.10	0.25	0.54	0.61
**Plasma CySS (µmol/L)**						
Mean change (95% CI)[e]	-4.08 (-6.11,-2.07)	-1.92 (-4.86,1.04)	-4.72 (-8.28,-1.16)	-2.46 (-8.10,3.18)	-3.93 (-6.80,-1.07)	-1.30 (-5.62,3.02)
p-value	<0.0001	0.20	0.01	0.39	0.007	0.55
**Plasma Cys Eh (mV)**[h]						
Mean change (95% CI)[e]	-2.62 (-4.78,-0.46)	-0.95 (-4.93,3.03)	-3.88 (-7.69,-0.07)	-4.75 (-12.03,2.52)	-2.11 (-5.14,0.93)	-2.04 (-7.82,3.73)
p-value	0.02	0.64	0.046	0.20	0.17	0.48

a – adjusted for log transformed age, sex, television ownership(yes/no), smoking (ever/never), log transformed body mass index, and GSH/Cys laboratory batch, b – additionally adjusted for log urinary creatinine, c –n=265 folate sufficient, d - n=111 folate deficient, e - represents the mean change in the outcome for a change in the exposure from the 25th to the 75th percentile, f – represents the ratio of the geometric mean in the outcome for a change in the exposure from the 25th to the 75th percentile, g - reduction potential of the GSH/GSSG redox pair, h – reduction potential of the Cys/CySS redox pair
